# Impacts of Stress Relief Treatments on Microstructure, Mechanical and Corrosion Properties of Metal Active-Gas Welding Joint of 2205 Duplex Stainless Steel

**DOI:** 10.3390/ma13194272

**Published:** 2020-09-25

**Authors:** Xiao-qin Zha, Yi Xiong, Tian Zhou, Yong-feng Ren, Peng-hui Hei, Zhi-liang Zhai, Jukka Kömi, Marko Huttula, Wei Cao

**Affiliations:** 1 Luoyang Ship Material Research Institute, Luoyang 471023, China; xiaoqinzha@sina.com; 2 Henan Key Laboratory of Technology and Application of Structural Materials for Ships and Marine Equipment, Luoyang 471023, China; 3 School of Materials Science and Engineering, Henan University of Science and Technology, Luoyang 471023, China; zhoutianv@163.com (T.Z.); 18262075136@163.com (Y.-f.R.); Marko.Huttula@oulu.fi (M.H.); 4 Luoyang Sunrui Special Equipment CO., LTD, Luoyang 471000, China; heipenghui@163.com (P.-h.H.); zhai_zl@163.com (Z.-l.Z.); 5 Materials and Mechanical Engineering, Center for Advanced Steels Research, University of Oulu, FIN-90014 Oulu, Finland; Jukka.Komi@oulu.fi; 6 Nano and Molecular Systems Research Unit, University of Oulu, FIN-90014 Oulu, Finland; Wei.Cao@oulu.fi

**Keywords:** 2205 duplex stainless steel welded joint, stress relief treatment, microstructure, mechanical properties, corrosion resistance

## Abstract

Stress relief treatments were carried out separately with a pneumatic chipping hammer, ultrasonic peening treatment, and heat treatment for metal active-gas welding (MAG) welded joints of 2205 duplex stainless steel. The effects of these methods on the residual stress, microstructure, mechanical properties and corrosion resistance of welded joints were studied. Results show the stress state of the weld and the surrounding area was effectively improved by the pneumatic chipping hammer and ultrasonic peening treatment, and the residual stress field of the surface layer changed from tensile stress to compressive stress. On the contrary, low-temperature stress relieving annealing had no obvious effect on stress distribution. After the pneumatic chipping hammer and ultrasonic peening treatment, the welded joints were machined and hardened. Correspondingly, strength and hardness were improved. However, the heat treatment only led to a slight decrease in strength and hardness due to the static recovery of the welded joint structure. All stress relief methods effectively improved the corrosion resistance of welded joints, with the ultrasonic peening treatment giving the best performance.

## 1. Introduction

As a third-generation duplex stainless steel (DSS), 2205 DSS is endowed with excellent welding performance, high toughness resulting from austenite, strong resistance to chloride corrosion and stress corrosion from ferrite [1,2,3]. Therefore, 2205 DSS has been widely used in the fields of energy, chemicals, construction, fossil fuels and transportation. Welding is an important part of the industrial processing of DSS pipes, flats and storage containers. However, the microstructure and mechanical properties of the welded joints differ from these of the base material. The quality of the welded joint directly determines the durability of the workpiece. A suitable welding method can significantly improve the quality of welded joints. Compared with electrode arc welding and submerged arc welding, metal active-gas (MAG) [4,5] welding has higher welding quality due to the action of the shielding gas. In the process of MAG welding, the heat generated by the arc is concentrated, and the range of the molten pool and heat-affected zone is small, which results in fewer defects. Lopes et al. [6] studied the influence of the MIG/MAG welding process on mechanical and pitting corrosion behaviors on SAF 2507 steel welded joints. Results show the increased amount of γ-phase improved the pitting corrosion resistance mainly in the molten zone. The toughness and hardness in the molten zone increased with increased formation of γ-phase. García-Rentería et al. [7] studied the resistance to localized corrosion of AISI 2205 steel joined by MAG welding under the effect of electromagnetic interaction. Results show the modified microstructural evolution of welded joints induced by application of electromagnetic interaction during welding is associated with the increase of resistance to localized corrosion of the welded joints. Welded joints made by MAG using the shielding gas 97% Ar + 3% N_2_ without the application of a magnetic field presented high resistance to general corrosion but high susceptibility to localized attack. MAG welding has been widely used in the production of duplex stainless steel and good results have been obtained.

Welding introduces many unknown factors to the joint and base material. In addition to ratio variation of the austenite and ferrite phases, compounds precipitation (σ, χ, Cr_23_C_6_, Cr_2_N, etc.), slag inclusion, and blowholes turn out [8,9,10] and result in a large residual tensile stress at the welded region. Thermal stress is the main part of residual stress, which is caused by the resolidification and cooling shrinkage of weld material after melting. Due to the difference of the chemical composition between the weld material and the base material, the structural stress caused by the phase transition during the cooling process is also an important part of the residual stress [11,12]. Thus, the welded joints are liable to crack, accompanied by rather poor corrosion resistance and low fatigue life [13]. Stress relief treatments are desired to improve residual stress states at weld seams and enhance joint performances. Cho et al. [14] studied residual stress relaxation characteristics under cyclic loading of the welded DSS pipes via finite element numerical simulation. Results show residual stresses may partially or completely be released during alternating loading. Under cyclic loading, welding residual stresses are inclined to relax to some extent due to cyclic plastic straining and reach a stabilized state after a certain number of cycles if cyclic plasticity is present. Gideon et al. [15] investigated intergranular corrosion performance and residual stress distribution characteristics of seams of the weld DSS pipeline. It was found that the tensile strain existed in both ferrite and austenite phases of the welded joint. Intergranular corrosion did not directly correlate to the strain distribution in the welding area. Alam et al. [16] studied relationships between residual stress and the microstructure of martensitic stainless steel during laser cladding. One-hour heat treatment at 565 °C significantly reduced the tensile stress on the surface and subsurface and homogenized the compressive stress throughout the bead and dilution zones.

Despite aforementioned efforts to unveil connections between stress distribution and microstructure of welded joints for stainless steels, correlations between microstructure evolutions and properties of welded DSS joints under different stress relief treatments are not well investigated. Herein, influences of stress removal methods on microstructures and performances were systematically studied for MAG welded joints of 2205 DSS. Three methods were employed, namely a pneumatic chipping hammer, ultrasonic peening treatment and heat treatment. The pneumatic chipping hammer has been widely used in industrial production because of its simple operation. In this process, hammer force, hammer frequency and hammer temperature have a great influence on the elimination of residual tensile stress of welded joints. Large residual compressive stress can be formed on the surface of the welded joint after hammering, which is beneficial to improve the fatigue life. As a new treatment method, ultrasonic shot peening can obtain more uniform residual compressive stress layer. Its better controllability also extends its application in the field of welding. A traditional stress-relieving heat treatment method is the experimental contrast. The effects of these methods on the MAG welded joints of 2205 duplex stainless steel is significant, which can provide technical support for its industrial application.

## 2. Experimental Procedures

### 2.1. Experimental Material

Commercial 2205 duplex stainless steel with a thickness of 6 mm was selected as the research object. MAG welding of 2205 duplex stainless steel was performed by using ER2209 solid wire with a diameter of 1.2 mm. The chemical compositions (wt.%) of 2205 duplex stainless steel and solid wire are listed in Table 1.

### 2.2. Experimental Process

Automated MAG welding of 2205 duplex stainless steel plate was carried out via a three-layer welding process in an inert ambience filled with 98% Ar + 2% N_2_ gases. A backing bar was used during the welding process. The welding current was 160~220 A, voltage 27 V, welding speed 100~160 mm/min, gas flow rate 12~15 L/min, and contact tip to work distance (CTWD) 15 mm. Before welding, a “V” groove with an angle of 60° was made on the joint of two steel flats. The welded specimens were divided into four groups. No.1 was the untreated specimen where no stress relief treatment was conducted during and after welding. When the interlayer temperature was less than 100 °C, No. 2 and No. 3 were specimens subjected to the pneumatic chipping hammer and ultrasonic peening treatment, respectively. The air pressure of the pneumatic chipping hammer was 0.6 MPa and the frequency was 2200 b/min. During the ultrasonic peening treatment, the tip pin diameter was 3.0 mm with an impact velocity of 67~200 mm/min, frequency of 16 kHZ, and impact range covering the full weld and 2~3 mm toe off the base material. No. 4 was the specimen subjected to stress relief treatment at 250 °C × 24 h after welding. A low-temperature, long-time heat treatment process is to eliminate the residual stress better on the premise of ensuring the microstructure does not change, which is common in industrial production. In addition, the treatment temperature is close to the service condition temperature in reality. Thus, we selected such a heat treatment process.

Microstructure observation was carried out for four groups of specimens in different treatment states. The etchant was sodium sulfite hydrochloric acid solution. The base material, welding line, and heat-affected area of each group of specimens were observed on a ZEISS Z1M (Jena, Germany) optical microscope. The ferrite content was determined through Micro-image Analysis and Process (MIAPS) metallographic quantitative analysis software (version 2.1) and measured in at least 10 photos. In addition, a 10 × 10 × 0.5 mm slice specimen was cut along the weld axis. The slice specimen was mechanically grinded to about 40 μm and polished, then rushed out to a Φ 3 mm foil. The foil was thinned on a Gatan 691 (Pleasanton, CA, USA) thinning instrument until a small hole turned out at the center of the slice specimen. To gain more information of prepared specimen microstructures, a JEM-2010 (Tokyo, Japan) transmission electron microscope (TEM) was used for observation at a working voltage of 200 kV.

**Figure 1:**
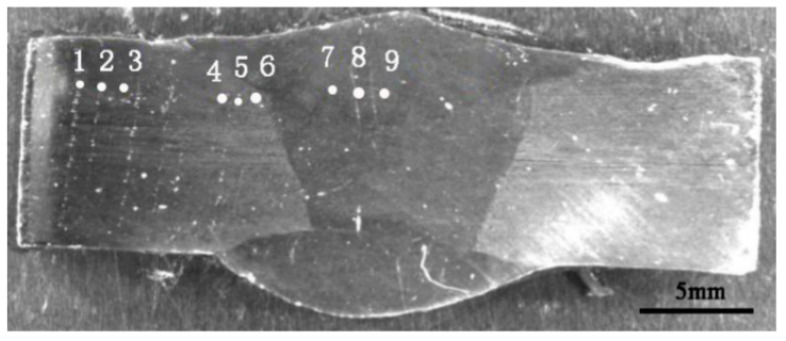
Microhardness point location.

**Figure 2:**
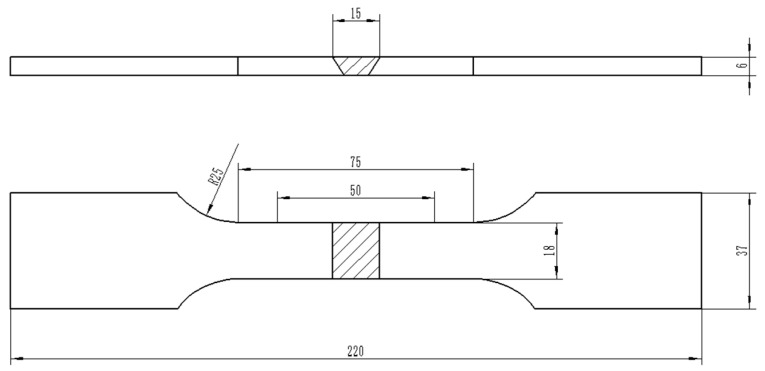
Schematic diagram of flat tensile specimen.

Hardness of each specimen group under different treatment states was tested with a MH-3 microhardness tester (Minsks, Xian, China). Locations of test points are shown in Figure 1. The load was 10 kg, and the loading and retention times were set to 10 s. The hardness of each group of specimens was measured three times at different locations and then averaged. The sampling position of the tensile specimen was perpendicular to the direction of the welding line, and the weld joint was located in the middle of the tensile specimen. Figure 2 shows the size and morphology of the tensile specimen. Subsequently, the mechanical properties of the specimens were tested on an Instron5587 (Boston, MA, USA) tensile testing machine at a loading speed of 0.5 mm/min. At least three tensile specimens were tested in each group to ensure reliability.

An XStress3000 G3 X-ray stress meter (Stresstech Oy, Tikkutehtaantie, Finland) was employed to detect residual stresses of specimens under different treatment states. The test parameters were: MnKα ray, γ-Fe(311) diffraction crystal plane, 30 kV accelerating voltage, 6.6 mA accelerating current, 10 s exposure time. When residual stress is present, the interfacial spacing of the specimens will change. In Bragg diffraction, the diffraction peak will also move, and the distance of the shift is related to the stress. An X-ray with wavelength λ was irradiated to the specimens at different incident angles several times successively, resulting in a corresponding diffraction angle 2*θ*. The angle between the normal of the diffraction crystal plane and the normal of the specimen surface is *ψ*. We substituted the slope of 2*θ* with respect to sin^2^*ψ* into formula 1 to find the stress value *σ*. The residual stress was noted as *σ_X_*, along the welding line, and as *σ_Y_*, perpendicular to the welding line.

(1) $$\sigma =-\frac{E}{2(1+\upsilon )}\cot\theta_{0}\frac{\pi }{180}\frac{\partial (2\theta )}{\partial (\sin^{2}\psi )}$$

where, *E* is Young’s modulus, *υ* is Poisson’s ratio, and *θ*_0_ is Bragg’s angle in the stress-free state.

An intergranular corrosion test was carried out on four groups of specimens in different treatment states. The specimens were continuously boiled in the 50% sulfuric acid-ferric sulfate solution for 12 h. According to the weight change (weight loss) of the specimen, the intergranular corrosion rate was calculated. The intergranular corrosion was also characterized through photographs of the polished sections. A Gill AC Bi-STAT electrochemical workstation was used to test the electrochemical corrosion performance of specimens in each group. To accelerate corrosion rates and keep the same anionic type as in the commonly used NaCl, the FeCl_3_ was selected as the corrosive medium due to the well-known high oxidative activity of the Fe^3+^. The solution was fixed to 6% FeCl_3_ and the experimental temperature was 25 °C. A three-electrode system was used for the test: a saturated calomel electrode was the reference electrode, a platinum electrode was the auxiliary electrode and the specimen was the working electrode. First, the open-circuit potential was measured for 60 min, and then the polarization curve was measured using the potentiodynamic polarization method. The scanning rate was 20 mV/min, the scanning started from −150 mV (versus Open Circuit Potential) to 1.5 V (versus Saturated Calomel Electrode). If the current density exceeded 10 mA/cm^2^ during the scanning of this potential interval, the scanning was stopped immediately. This is because when the current density exceeds 10 mA/cm^2^, the specimens can be considered to have been seriously corroded.

## 3. Results

### 3.1. Residual Stress Analysis

**Figure 3:**
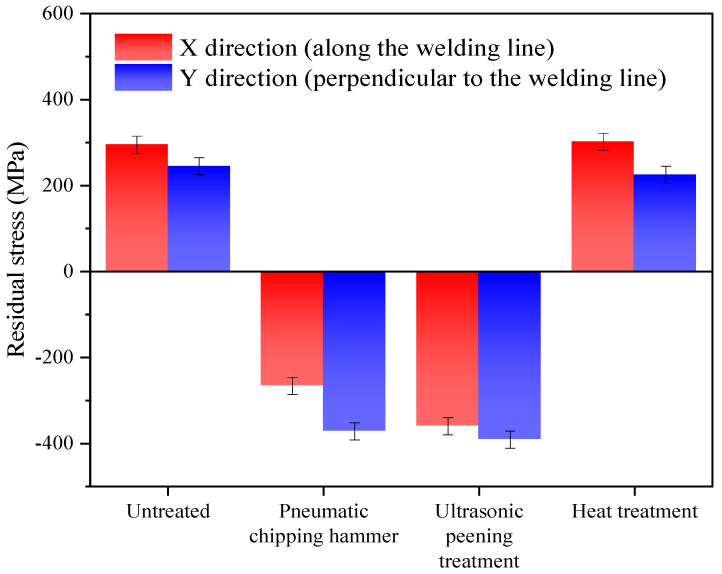
Residual stress of welded joints with different treatments.

Figure 3 depicts residual stresses of 2205 duplex stainless steel welded joints under different treatment states. From Figure 3, surface residual stresses in both the X and Y directions of specimen 1 are tensile stress. High surface residual tensile stress will accelerate the failure of the workpiece and lead to the decline of fatigue life [18,19]. Specimens 2 and 3 showed compressive stress along the X direction and Y direction. Residual compressive stress on the surface can inhibit adverse effects of tensile stress, slow down the initiation and propagation of cracks, and improve the fatigue life of the workpiece [20,21]. Compared with specimen 1, stress changes of the X-direction and Y-direction in specimen 2 were 560 MPa and 617 MPa respectively. Similarly, the values were 654 MPa and 636 MPa for specimen 3. Both the pneumatic chipping hammer and ultrasonic peening treatment are beneficial for diminishing tensile stresses. However, specimen 4 was kept in the tensile stress state. The tensile stress in the Y direction was the same as that before heat treatment, and the tensile stress in the X direction even increased a little after heat treatment, which can be attributed to the measurement error. The above result shows that postweld heat treatment at 250 °C × 24 h had no obvious effect on improving the residual stress at the welded joint [22,23].

### 3.2. Metallographic Structure

**Figure 4:**
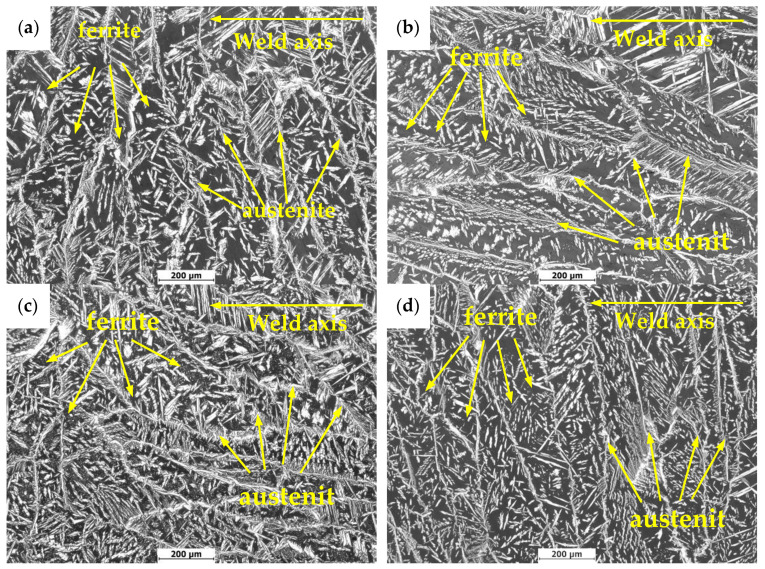
Microstructure of DSS 2205 weld seam area with different treatment methods: (**a**) untreated; (**b**) pneumatic chipping hammer; (**c**) ultrasonic peening treatment; (**d**) heat treatment.

Figure 4 shows the metallographic structures of weld seam areas in 2205 duplex stainless steel under different treatment states. In Figure 4, the austenite phases in all specimens’ welds can be divided into three categories: feathery austenite (weylanite austenite) diffused into ferrite along the grain boundary, austenite precipitated along the grain boundary of ferrite and austenite precipitated in the grain of ferrite [24]. During the welding process of 2205 duplex stainless steel, the microstructure at the welded joint first changes to high-temperature ferrite. Subsequently, austenite grains begin to nucleate at different locations of ferrite during cooling and form dendritic crystals at high cooling rates [25]. Under different treatment conditions, the microstructure and two phase content of the specimens were obviously different. In the untreated specimen 1, the ferrite and austenite of the weld joint grew perpendicularly to the welding line. Changes were noticed in the welded joints of specimen 2 and 3 compared with specimen 1. After the pneumatic chipping hammer and ultrasonic peening treatment, the ferrite and austenite were elongated parallel to the welding line. In the process of treatment, due to the different microstructure of the weld area from that of the base material, the applied external stress tended to induce plastic deformation of two-phase along the direction parallel to the welding line, resulting in the residual compressive stress in the Y direction. Extension of the two-phase microstructure turned out in the direction parallel to the welding line.

**Figure 5:**
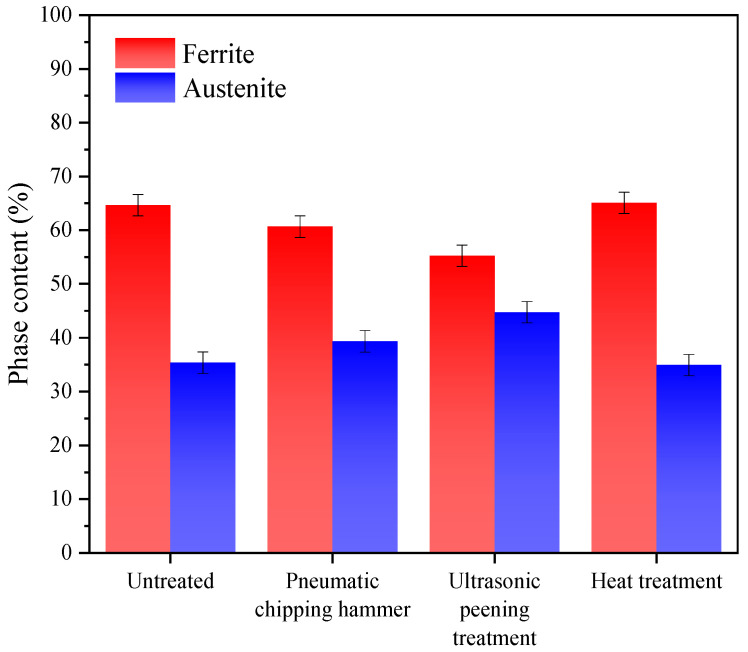
Ferrite and austenite content of DSS 2205 weld seam area with different treatments methods.

Figure 5 shows the test results of two-phase content in the weld area of 2205 duplex stainless steel under different treatment states. In Figure 5, the austenite contents of specimens 2 and 3 are higher than that of specimens 1. The content of specimens 3 significantly increased from 35.37% to 44.76%. This is because, in welding, all ferrite is present at the beginning of solidification at high temperatures. When cooled to 1300 °C, austenite begins to nucleate at the ferrite grain boundaries or within grains. In the process of the pneumatic chipping hammer and ultrasonic peening treatment, although the ferrite to austenite transformation will not occur due to deformation induction at low temperature, a large number of crystal defects, such as dislocation, will be introduced into the ferrite. These defects provide a large number of nucleation sites for the subsequent formation of austenite, resulting in a higher austenite volume fraction than specimen 1 [26,27]. Compared with specimen 1, specimen 4 did not show significant changes in microstructure and ferrite content, which also indicates that the heat treatment at 250 °C × 24 h did not induce the microstructure transformation.

**Figure 6:**
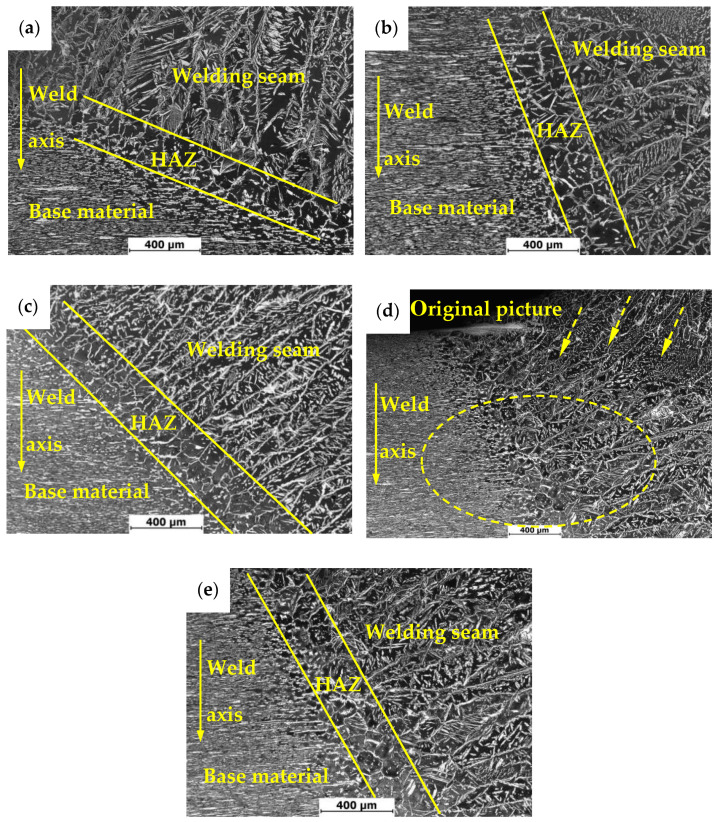
Metallographic structure of DSS 2205 heat-affected zone with different treatment methods: (**a**) untreated; (**b**) pneumatic chipping hammer; (**c**) ultrasonic peening treatment; (**d**,**e**) heat treatment.

Figure 6 shows metallographic structures of heat-affected zones in 2205 duplex stainless steel under different treatment states. Microstructures of heat-affected zones are obviously different from that of the base material and weld seam area. In fact, microstructure of the heat affected zone was formed by rapid heating and cooling processes of the base material. Such a formation of the microstructure is a non-equilibrium transformation process, resulting in its far deviation from the equilibrium structure [28]. As can be seen from Figure 6, the orientation of weld microstructure of specimen 1 is perpendicular to the base material, while that of specimen 2 and specimen 3 is nearly parallel to the base material, which is consistent with the results in Figure 4. In Figure 6e, the orientation of the weld microstructure of specimen 4 is nearly parallel to the base material, which is mainly related to the cropping of the picture. As can be seen from the original picture (Figure 6d), the microstructure orientation of the welding seam in the upper right corner is almost perpendicular to the base material, which also confirms the conclusion in Figure 4.

**Figure 7:**
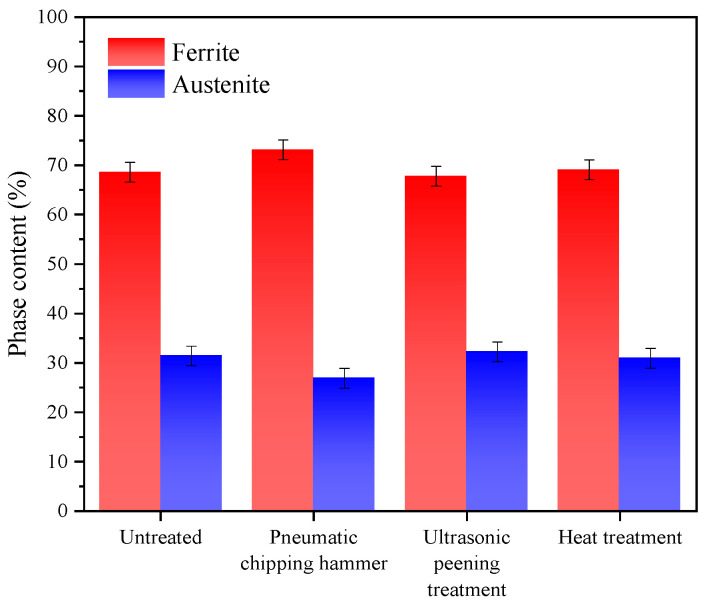
Ferrite and austenite content of DSS 2205 heat-affected zone with different treatment methods.

Figure 7 shows test results of two-phase content in the heat-affected zone. Compared with the weld seam area, the ferrite content in the heat affected zone increases significantly. The ferrite content gets to a highest value of 73.1% in specimen 2 and becomes slightly lower in specimen 3 (67.8%). The ferrite contents in specimen1 and specimen 4 are similar (69%). Due to the thermal cycling process of the heat affected zone, the austenite was transformed into ferrite, and the ferrite grains grew significantly, which makes the ferrite content in the heat affected zone reach the highest level in the whole material [28,29]. After a lot of repeated experiments, it is found that the ferrite content in the heat-affected area of the specimens treated by pneumatic chipping hammer is slightly higher than that of the specimens treated by ultrasonic treatment. This may mostly due to (a) larger deformation rate from hammering than mechanical wave, and (b) locally higher temperature induced by hammering than phonon concave. However, to precisely determine deformation dynamics and accessory heating load requires very dedicated setups and instrumentations. At the present experiments, we only paid attention to the ferrite content with a good corresponding relationship with hardness distribution, but neglected the reasons for the increase of ferrite content in this region. In the subsequent work, we will focus on the reasons for the increase of ferrite content caused by pneumatic chipping hammer.

### 3.3. TEM Observation

**Figure 8:**
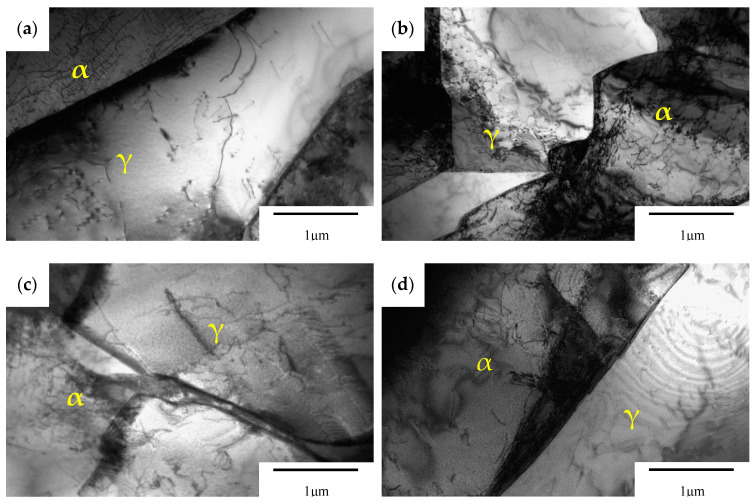
TEM-observed microstructure of DSS 2205 welded joints with different treatment methods: (**a**) untreated; (**b**) pneumatic chipping hammer; (**c**) ultrasonic peening treatment; (**d**) heat treatment.

Figure 8 shows the TEM-observed microstructure of the welded joints in 2205 duplex stainless steel under different treatment states. No precipitated phase was observed in the austenite, ferrite and austenitic-ferrite phase boundary of all specimens. Microstructures of all specimens differed in dislocation distributions. In specimen 1, the dislocation density in austenite and ferrite was low, and the dislocation was mainly distributed at the boundary of α/γ phases. On the contrary, more dislocations were found in both austenite and ferrite of specimens 2 and 3. The dislocation density in ferrite was significantly higher than that in austenite. This is because ferrite’s stacking fault energy is higher than austenite’s. In the process of surface plastic deformation, a large number of dislocations are more likely to form in ferrite, resulting in dislocation proliferation and entanglement [30,31]. Dislocation densities of ferrite and austenite were lower in specimen 4 than in specimen 1. This is due to the dislocation rearrangement and annihilation caused by the recovery phenomenon during stress relief annealing [32,33]. The TEM result is consistent with the test results of residual stress redistribution in the surface layer.

### 3.4. Mechanical Property

**Figure 9:**
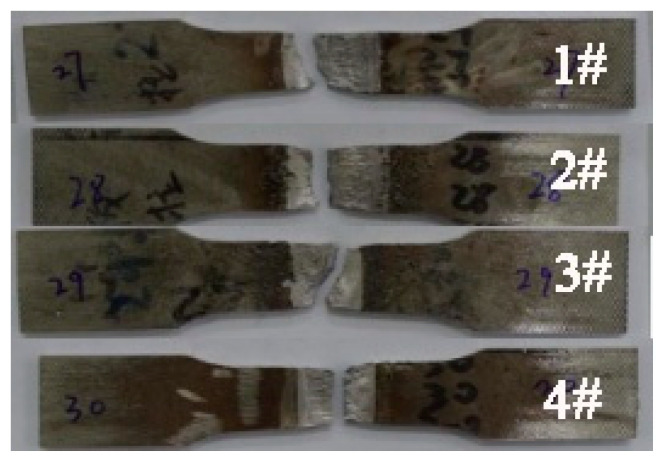
Fracture locations of flat tensile specimens with different treatments methods: 1—untreated; 2—pneumatic chipping hammer; 3—ultrasonic peeing treatment; 4—heat treatment.

**Figure 10:**
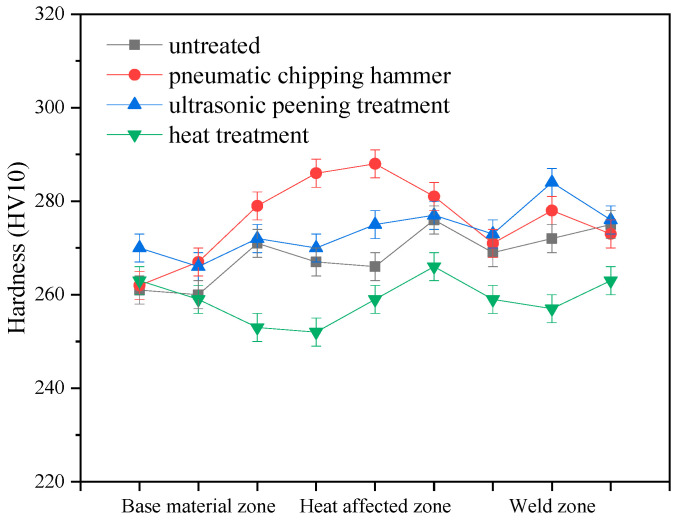
Hardness distribution of welded joints with different treatments.

Figure 9 shows fracture positions of 2205 DSS tensile specimens under different treatment states. All fractures are located at the weld seams, indicating that significantly lower strength at the weld than at the base material. The tensile strengths of specimens 1, 2 and 3 had similar values of 823 MPa, 825 MPa and 829 MPa, respectively, while the tensile strength of specimen 4 was the lowest at only 797 MPa. Figure 10 is the hardness distribution of welded joints subjected to different treatments. Compared with specimen 1, the hardness of specimens 2 and 3 was improved. This is because the pneumatic chipping hammer and ultrasonic peening treatment have obvious work hardening effects on the surface of organization. Plastic deformation increases the dislocation density in the microstructure, which is prone to dislocation entanglement and plugging, resulting in the increase of material hardness. However, the hardness of specimen 4 was slightly lower after treatment. This is attributed to static recovery and dislocation rearrangement annihilation that occurred in the organization during low-temperature annealing. Overall strength and hardness of the material were reduced after microstructural evolution.

### 3.5. Corrosion Resistance

#### 3.5.1. Resistance to Intergranular Corrosion

**Figure 11:**
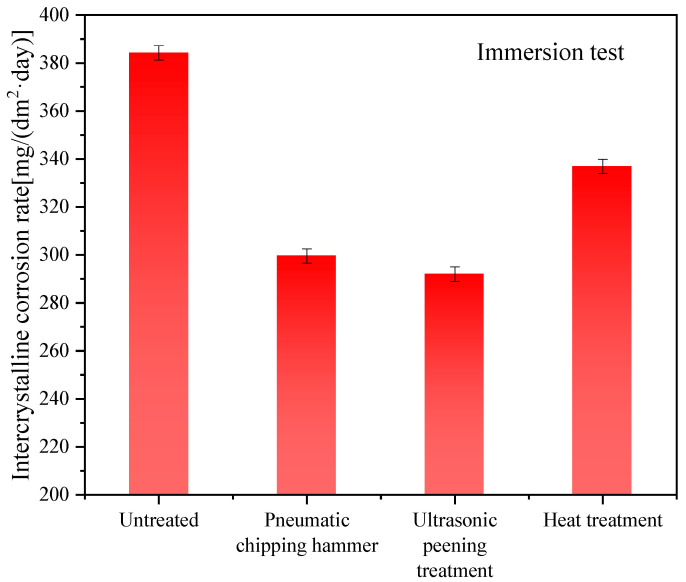
Intergranular corrosion rates of welded joints with different treatments.

**Figure 12:**
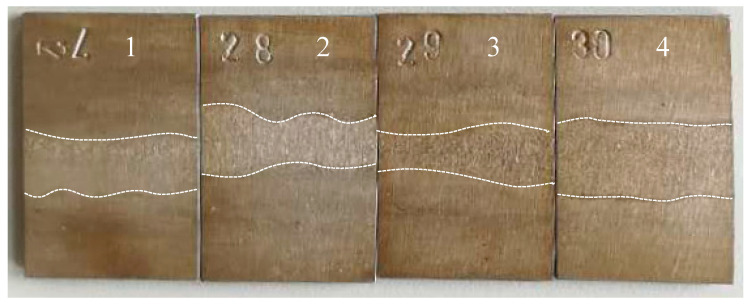
Images of intergranular corrosion welded joints with different treatments methods: 1—untreated; 2—pneumatic chipping hammer; 3—ultrasonic peening treatment; 4—heat treatment.

**Figure 13:**
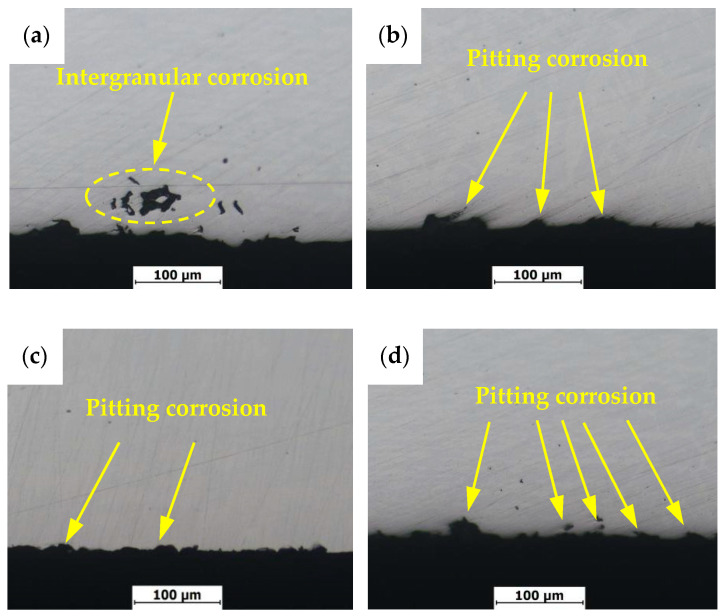
Cross section morphology of intergranular corrosion welded joints with different treatments methods: (**a**) untreated; (**b**) pneumatic chipping hammer; (**c**) ultrasonic peening treatment; (**d**) heat treatment.

**Figure 14:**
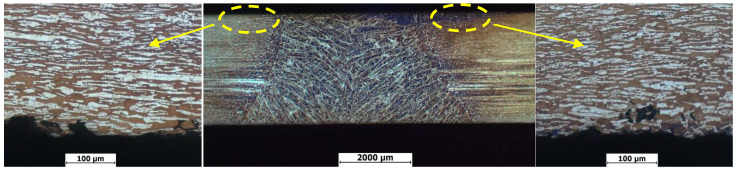
Cross section morphology of untreated sample after corrosion test.

Figure 11 shows intergranular corrosion rates of welded joints in 2205 DSS under different treatment states, in which the unit of corrosion rate is mg/(dm^2^·day), abbreviated as mdd. The intergranular corrosion rates were significantly reduced after stress relief treatments, indicating all stress relief treatments are beneficial to the intergranular corrosion resistance of the welded joint. Figure 12 shows macroscopic features of the welded joints after intergranular corrosion. After the intergranular corrosion tests, obvious strips turned out at the weld seam and heat-affected zone in the middle of the specimens as a result of compositional and microstructural differences. To further determine the locations of intergranular corrosion, corroded specimens were cut from the intermediate vertical welding line and the cross-section microstructures were observed. As shown in Figure 13, at a certain distance from the surface, point, strip and ring corrosion appear on the subsurface of specimen 1 (see Figure 13a). Therein, the grain boundary between the subsurface and unpolished surface provided a medium to host intergranular corrosion in specimen 1. However, the specimens after the stress relief treatments only formed pits on the surface, showing pitting corrosion characteristics (see Figure 13b–d). Therefore, it can be concluded that specimens 2, 3 and 4 only exhibited pitting corrosion, but not intergranular corrosion. In order to prove that the holes in the metallography were caused by corrosion, corresponding verification tests were carried out, as shown in Figure 14. After etching specimen 1 after intergranular corrosion, it was found that the holes were not in the weld zone, but in the base metal zone near the heat affected zone. Therefore it is very certain that it was not a welding defect but was caused by corrosion. It could be further seen from the enlarged metallographic photos that corrosion occurred along the grain boundary. On the other side of specimen 1 near the heat affected zone, we also found this phenomenon. This indicates that there will be intergranular corrosion in the base metal near the heat affected area of the untreated welded joint, but after stress relief treatments, the effect is much smaller or can be avoided.

#### 3.5.2. Electrochemical Corrosion Resistance

**Figure 15:**
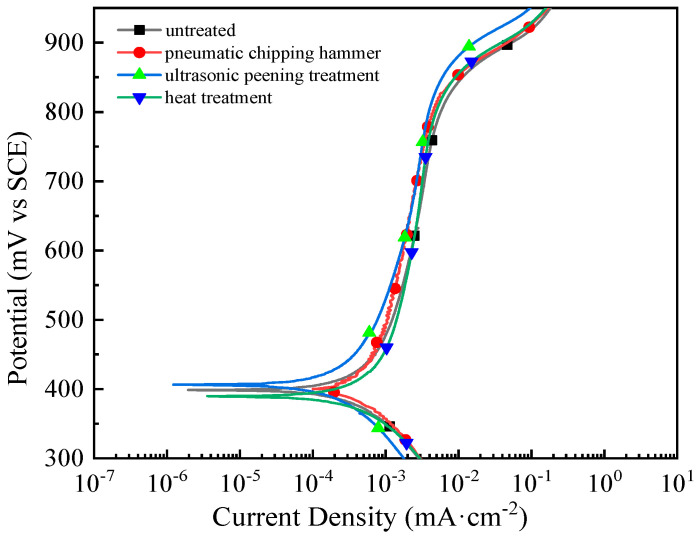
Polarization curve of the welded joints with different treatments methods: 1—untreated; 2—pneumatic chipping hammer; 3—ultrasonic peening treatment; 4—heat treatment.

Figure 15 shows polarization curves of the welded joints with different treatments, and the electrochemical parameters are tabulated in Table 2. Corrosion potentials of welded joints are relatively similar after all stress relief treatments. However, corrosion current densities were reduced to some extent, indicating significant improvement of the corrosion resistance of welded joints after the treatment. The result is also consistent with those from the above intergranular corrosion tests (see Figure 11, Figure 12 and Figure 13). Among all specimens, specimen 3 had the lowest corrosion current density, denoting the lowest corrosion rate and the best corrosion resistance. Specimen 2 had a slightly worse resistance. In comparison, specimen 4 had only a small decrease in current density. The above observation can be explained as follows. After the pneumatic chipping hammer and ultrasonic peening treatments, the residual stress state changed from tensile to compressive stress, and austenite content significantly increased. As a combined impact from the phase and stress types, corrosion resistance was enhanced [34,35]. After a long time of low temperature annealing treatment, although the macroscopic structure of the welded joint had no obvious change, the dislocation rearrangement annihilation in the microscopic structure reduced the number of structural defects. This improved corrosion resistance of the welded joint but only to a limited extent. Pitting potential reflects the difficulty of surface passivation film to be broken down. When the voltage reaches the pitting potential, the passivation film on the surface of the material breaks, leading to violent corrosion. The pitting potential of sample 3 was the highest, indicating that it has the best corrosion resistance.

## 4. Conclusions

In this paper, the microstructure, mechanical property and corrosion resistance of 2205 duplex stainless steel MAG (2% N_2_ + 98% Ar protective gas) welded joints under three stress relief treatments were studied. The main conclusions are as follows.

1. After the pneumatic chipping hammer and ultrasonic peeing treatment, the stress states of the welded joints were effectively improved from residual tensile stress to residual compressive stress. The stress values along the weld direction and perpendicular to the weld direction of the specimens treated by the pneumatic chipping hammer were 560 MPa and 617 MPa respectively, and those of the specimens treated by ultrasonic peening were 654 MPa and 636 MPa respectively. The ultrasonic peening treatment proved to be the best option. On the contrary, low-temperature stress-relieving annealing had no obvious effect on stress distribution.
2. Pneumatic chipping hammer and ultrasonic peening treatment can change the weld surface of two-phase grain growth direction, from vertical growth to growth parallel to the weld axis. Furthermore, dislocation density in the two-phase microstructure increases. The average strengths of specimens treated by pneumatic chipping hammer and ultrasonic peening were close to that before treatment, 825 MPa and 829 MPa respectively. Furthermore, the hardness of specimens treated by pneumatic chipping hammer and ultrasonic peening treatment increased slightly, to about 280 and 270 respectively. For stress relief annealing specimens, static recovery occurred, dislocation density decreased and no second phase precipitation occurred. The welded joint strength decreased to 797 MPa and hardness decreased to about HV 260.
3. The pneumatic chipping hammer, ultrasonic peening treatment and stress relief annealing treatment can effectively improve corrosion resistance of welded joints, and the intergranular corrosion rates decrease from 384.22 mdd to 299.58 mdd, 291.95 mdd and 336.86 mdd, respectively.

## Figures and Tables

**Table 1:** Chemical composition of 2205 duplex stainless steel and solid wire (wt.%) [17].

Material	C	Si	Mn	Cr	Ni	Mo	N	Fe
2205	0.025	0.60	1.50	22.50	5.70	3.00	0.15	Balance
ER2209	0.017	0.57	1.61	22.06	8.84	2.68	0.11	Balance

**Table 2:** Experimental results of electrochemistry of welded joint with different treatments.

Processing Method	Corrosion Potential (mV)	Corrosion Current Density (mA/cm^2^)	Pitting Potential (mV)
Untreated	398	7.3 × 10^−4^	821
Pneumatic chipping hammer	400	6.3 × 10^−4^	856
Ultrasonic peening treatment	407	4.6 × 10^−4^	895
Heat treatment	389	7.1 × 10^−4^	852
